# Colonoscopic Polypectomy

**DOI:** 10.1155/DTE.6.111

**Published:** 2000

**Authors:** Jerome D. Waye

**Affiliations:** 1 Mount Sinai Medical Center; GI Endoscopy Unit Mount Sinai Hospital GI Endoscopy Unit Lenox Hill Hospital USA; 2 650 Park Avenue New York NY 10021 USA

## Abstract

Colonoscopic polypectomy is a major advance in the therapy of colon neoplasms. The techniques for safe and efficient polyp removal are described. The uses of a variety of ancillary devices are discussed, including clips, loops, submucosal injection of fluid, and several thermal probes, including the argon plasma coagulator. The location of a lesion may be difficult to ascertain by intracolonic landmarks, but can be more precisely determined by X-ray, magnetic imaging, or intraoperative colonoscopy. Alternatively, it is possible to permanently mark the site of polyp removal with a carbon particle submucosal injection to facilitate subsequent localization either by surgery or interval colonoscopy.


*Diagnostic and Therapeutic Endoscopy*, Vol. 6, pp. 111–124
Reprints available directly from the publisher
Photocopying permitted by license only

© 2000 OPA (Overseas Publishers Association) N.V.
Published by license under
the Harwood Academic Publishers imprint,
part of The Gordon and Breach Publishing Group.
Printed in Malaysia.

### Colonoscopic Polypectomy

JEROME D. WAYE*

*Mount Sinai Medical Center; GI Endoscopy Unit, Mount Sinai Hospital; GI Endoscopy Unit, Lenox Hill Hospital*

*(Received 4 October 1999; Revised 7 December 1999; In final form 21 December 1999)*

**Colonoscopic polypectomy is a major advance in the therapy of colon neoplasms. The techniques for safe and efficient polyp removal are described. The uses of a variety of ancillary devices are discussed, including clips, loops, submucosal injection of fluid, and several thermal probes, including the argon plasma coagulator. The location of a lesion may be difficult to ascertain by intracolonic landmarks, but can be more precisely determined by X-ray, magnetic imaging, or intraoperative colonoscopy. Alternatively, it is possible to permanently mark the site of polyp removal with a carbon particle submucosal injection to facilitate subsequent localization either by surgery or interval colonoscopy.**

*Keywords:* Marking, Problems, Safety, Submucosal injection, Technique

### INTRODUCTION

The removal of colonic polyps is a major advance in medicine. Polypectomy was first introduced thirty years ago, and it has markedly changed the way polyps and cancer are treated. Polypectomy is safe and is currently the only (but not the absolute) method for prevention of colon cancer by interrupting the polyp-cancer sequence.

### EQUIPMENT

### Electrosurgical Unit

Most experts use pure coagulation current during polypectomy, and eschew blended current, which tends to separate a polyp more rapidly than does coagulation alone. Once adjusted to the optimal setting, there is no need to change the power output during polyp removal, regardless of whether the base is large or small or when switching between the polypectomy snare and the hot biopsy forceps.

### Endoscopes

A single-channel 168 cm-long colonoscope with a large accessory channel is the instrument most preferred for colonoscopy by all experts and most colonoscopists. The double-channel scopes are somewhat less flexible, can be difficult to pass through the entire colon, and are associated with more patient discomfort than the one-channel type.

* Address for Correspondence. 650 Park Avenue, New York, NY 10021, USA. Tel.: +1-212-439-7779. Fax: +1-212-249-5349. E-mail: jdwaye@aol.com.

There are only limited occasions when it is desired to pass two accessory devices simultaneously through a colonoscope, such as grasping a polyp and lifting it while placing a snare [1–3]. This maneuver would appear to be relatively easy, but in practice can be quite difficult, since the two accessories are obligated to move together rather than separately; it is desirable to lift up the portion grasped by the forceps while seating the snare downward over the polyp, but such manipulation is not possible.

### Hot Biopsy Forceps

The hot biopsy forceps is an electrically insulated forceps through which electrical current flows to direct electrical energy around the tissue held within the jaws, enabling simultaneous cautery of a polyp base while obtaining a biopsy specimen.

### Thermal Devices

Two contact thermal devices, the heater probe and Bicap electrode, can control post-polypectomy arterial spurting as well as lesser degrees of hemorrhage. When used for control of colonic hemorrhage, the current should be decreased by approximately 50% from the power used for treatment of upper intestinal hemorrhage. Multiple applications at a low power setting appear to be safe in the colon. There should not be a great deal of force as is recommended in the upper intestinal tract. The water jet from these probes is extremely useful for precise localization of the bleeding site to permit precise probe application.

The argon plasma coagulator is a device for the delivery of high-frequency current in a monopolar mode that does not require direct contact between the probe and tissue. When the distance from probe to tissue is optimal, the monopolar circuit is completed by the flow of electrons through the activated and ionized argon gas which transmits the electrical ions from the probe electrode to the tissue. The ionized argon gas is called the argon plasma. Utilizing a combination of voltage adjustment and motion of the probe tip, the thermal penetration of tissue can be varied from 1 to 3 mm. In the colon, the power output setting should usually be at 40 W, with a relatively low gas flow (0.8 L/min). Uses in the large bowel include treatment of post-polypectomy bleeding and ablation of residual adenomas following their piecemeal resection [4–6]. The delivery system for flexible endoscopy was developed in Germany (ERBE, Inc., Tubingen, Germany) [7,8].

### Loops and Clips

Two additional devices are available for polypectomy. These are the detachable snare loop and clips. The loop, used for hemostasis during or after polypectomy, is a nylon ligature that can be placed over a lesion (such as a pedunculated polyp) and tightened with a one-way silicone-rubber stopper, which prevents opening of the loop after it has been closed [7]. The fully assembled mechanism, with an accompanying over-sheath (to permit the soft nylon loop to pass through the instrument channel), is the same caliber as a snare. Once extruded from the end of the delivery system, the loop is maneuvered around the head of a polyp under direct vision and, after tightening, is separated from the insertion tube [9]. When attempting to encircle large polyps, the floppy nylon loop – not having the same tensile strength of a wire snare – may become caught in the polyp head, resulting in the inability to pass the loop completely over the polyp. If the loop becomes enmeshed in the polyp head, closure of the loop will result in tangential placement interfering with snare positioning. If the head is encircled successfully, it is necessary that the loop be placed on the pedicle far enough toward the colon wall to allow transection of the stalk above the loop with sufficient margin to ensure safety if the polyp contains invasive cancer. After placement close to the bowel wall, polypectomy is performed above the loop-ligature. Transection of the pedicle close to the loop may cause the loop to slip off with immediate bleeding as a complication. Placement prior to polyp removal may result in difficulty with snare placement because of the long "tail." Because immediate post-polypectomy bleeding only occurs in about 1% of cases [10], an alternate use of this device is to place it on the resected stalk to ensure hemostasis post-polypectomy, although tissue retraction may flatten the residual pedicle to the extent that the loop cannot be properly seated [11]. The loops spontaneously slough in 4–7 days, and endoscopy following detachment of the loops shows residual shallow ulcers. The loop and carrier device (HX-20) are manufactured by Olympus Inc.; the loop is available in two sizes, 2.5 and 4.0 cm.

Mucosal clips have been available for several years. These small clips are useful for temporary marking of lesions, to identify the distance reached by the colonoscope [12], and to mark the edges of tumors prior to expandable metal stent placement. The clips may also be used for hemostasis [13] (Fig. 1). The ability to rotate the clip permits more precise placement [14]. The clips are especially useful for bleeding from flat polypectomy sites [15]. They have also been used successfully to stop arterial bleeding from the severed stalk of pedunculated polyps. It is theoretically possible to apply a clip onto the base of a pedunculated polyp close to the bowel wall and snare the polyp above the clip using standard snare techniques [16]. It is important that the wire snare does not touch the metal clip, which may result in a burn of the colon wall. The clips spontaneously dislodge within several days, although some may remain for long periods. Both loops and clips may be used prophylactically in patients with disorders of coagulation either spontaneous

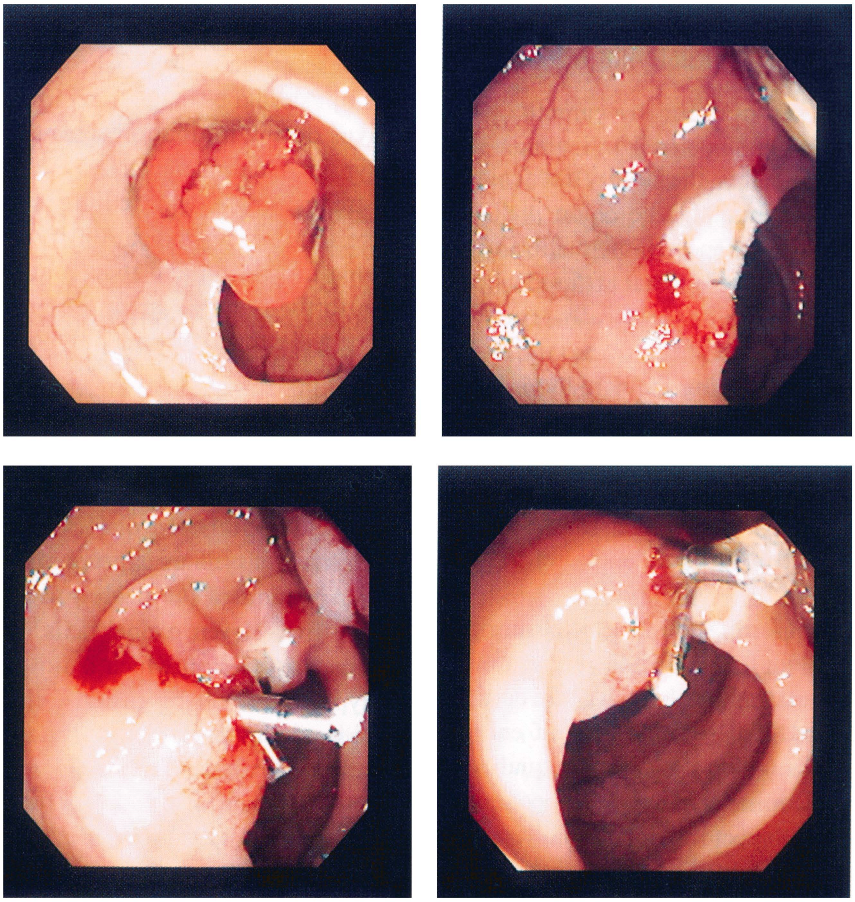

FIGURE 1 Clips placed on a bleeding site to control immediate post-polypectomy bleeding.

or iatrogenic (Coumadin) when there is a risk of subsequent bleeding.

### Snares

The standard large snare is about 6 cm in length by 3 cm wide, and the small snare is 3 cm long by 1 cm wide. The technique of application is the same in all instances, whether the snare is oval, crescent-shaped, or hexagonal. The diameter of the wire is an important consideration, since thin snare wires will cut through a polyp faster than a thick wire [17].

A bipolar snare [18] is available but does not have any benefit over the standard monopolar electrocautery snare. Rotatable snares are considered unnecessary by most endoscopists.

### PROCEDURE

### Safe Polypectomy

Safe polypectomy requires the ability to achieve hemostasis without damaging the colon wall. A successful polypectomy depends on achieving a balance between the two factors that are used whenever a polyp is removed colonoscopically. The two factors are heat, which causes cauterization, and the shearing force from tightening the wire loop. Both of the factors must be used to result in a clean, bloodless polypectomy without excessive burn to the colon wall. Heat alone will not cut through polyp, while guillotine force alone may sever a polyp, but can result in bleeding as blood vessels are not sealed by heat.

In general, the dial settings on the electrosurgical unit should be medium to low and the current may be either pure coagulation or blended. Pure cutting current is never used because this type of energy output explodes cells with no hemostatic qualities. Most expert endoscopists use only pure coagulation current for polypectomy. The delivery of energy should be continuous once polypectomy has commenced, and the person who closes the snare should do so slowly.

Patients with polyps of any size that can be removed endoscopically do not require hospitalization. Even the largest polyps may be removed totally, with or without submucosal injection polypectomy techniques, on an outpatient basis or in an ambulatory office setting.

### Polyp Size

The majority of polyps (about 80%) are relatively small, being less than 1 cm in diameter. All large polyps (greater than 35 mm) are adenomas and are usually located in the rectum or right colon. Most small (less than 5 mm) polyps in the rectum and distal sigmoid colon are non-neoplastic, but throughout the remainder of the colon, approximately 60–70% of small polyps are adenomas [19].

Fifty percent of patients with one adenoma will have another, and it is important to perform total colonoscopy to seek synchronous adenomas. No attempt should be made to remove large or difficult polyps during intubation since larger unresectable polyps or a malignancy may be encountered further upstream which will require surgery. However, there is no contraindication to removing a polyp during intubation and then continue the examination to the cecum despite the probability that the scope will rub against the polypectomy site.

### TECHNIQUE OF POLYPECTOMY

Accessories such as snares and forceps enter the viewing field at the five o'clock position, resulting in a relatively easy snare capture of a polyp in the right lower portion of the field, and occasionally those in the five–eleven o'clock axis. It is very difficult to ensnare a lesion which is not on that diagonal. Polypectomy will be easier if the instrument can be rotated to place the polyp in the right lower quadrant of the field of view.

### Small Polyps

Small polyps may be removed with the hot biopsy forceps, which provides a histologically identifiable tissue specimen, while electrocoagulation current ablates the polyp base in most instances [20–22]. To prevent deep thermal injury to the colon wall, the forceps should pull the polyp head away from the wall toward the colon lumen. As current is applied, a zone of white thermal injury will be seen at the base. When this injury zone is 1–2 mm wide, current should be discontinued and the specimen retrieved as with any biopsy technique. Because of reported complications with the hot biopsy forceps [23], many endoscopists only use them to eradicate polyps in the range 1–5 mm.

Small polyps in the range 1–4 mm have small nutrient blood vessels and can safely be removed by "cheese-wiring" with a mini-snare without electric current. This technique results in minimal bleeding [24,25]. The defect following cold snare excision resembles a punch biopsy site, with a disc of missing mucosa. Only rarely does bleeding occur, but this always stops within 1–5 min.

### Catheter Placement

The most important step in polyp capture is to place the catheter tip at the precise site where transection is desired after the snare loop has been placed over the polyp [17]. If the polyp is pedunculated, the tip of the sheath should be advanced to the mid-portion of the stalk. If sessile, the sheath tip should be advanced to the line of visible demarcation between adenoma and colon wall. Closure of the loop will result in seating the snare on the opposite side of the polyp, since the wire loop always concentrically closes toward the tip of the snare sheath, which is the fixed point in the polypectomy system.

### Pedunculated Polyps

A pedunculated polyp of any size should be able to be removed by a single transection [26]. Piecemeal resection of the head may be performed if the polyp cannot be encircled with the snare [27], until the residual portion of polyp is small enough and visualized sufficiently to permit encirclement of the pedicle with the wire loop.

### Sessile Polyps

The polyp with a wide attachment to the colon wall may be transected with one application of the wire snare, if the base is less than 1.5 cm in diameter. In the right colon, where the wall is thin, the endoscopist should consider piecemeal polypectomy or submucosal injection technique for any polyp whose base is over 1 cm. The heat produced by snare activation spreads toward the submucosa and serosa of the colon wall. The larger the polyp, the greater will be the volume of tissue captured within the wire loop, and a greater amount of thermal energy will be required to sever the polyp in the thin right colon. This may result in a full thickness burn of colon wall, which can result in a perforation of the bowel. The submucosal layers and muscularis propria together are only about 1 mm thick [28].

### Submucosal Injection Polypectomy (SIP)

The submucosal injection technique can be used for removal of sessile adenomas, whether small or large [29–31]. Injection of fluid into the submucosa beneath the polyp will increase the distance between the base of the polyp and the serosa. When current is then applied with a snare, the lesion can be more safely removed because of a large submucosal "cushion" of fluid. The fluid injected may be saline (normal or hypertonic) [32], with or without methylene blue to enhance visualization and with or without epinephrine [33]. Most endoscopists use normal saline only.

The needle may be placed into the submucosa just at the edge of a polyp, or if the polyp is large and flat, multiple injections may be given around the polyp or directly into the middle of the polyp. If a bleb does not form at the injection site when 1 ml of fluid has been given, the needle should be withdrawn since the tip may have penetrated the full thickness of the wall. A large localized fluid collection is the desired endpoint, with marked elevation of the polyp. If the needle placement is too superficial, the fluid will leak out and spill into the lumen. This is especially noticeable when a colored fluid is used, such as methylene blue or India ink. If possible, the approach by the needle injector should be tangential to the mucosal surface instead of perpendicular. The desired elevation of the polyp may take 3–4 ml of saline given in several places, although some authors use up to 30 ml of fluid [34]. Polyps up to 2 cm in diameter may be removed with one application of the snare, but larger polyps may require several piecemeal transections [35] (Fig. 2).

In general, malignant tumors should not be removed by the submucosal injection technique. If a polyp fails to elevate (the "non-lifting sign") [36], it may be an indication of infiltration by cancer into the submucosa, with tumor fixation limiting the expansion of the submucosal layer. Although deep or superficial needle placement may be the cause for failure to raise a bleb under a polyp, a submucosal bulging or bleb on one side of a polyp without any visible elevation of the tumor itself is a clue that there is fixation into the submucosa. This phenomenon may also be caused by a prior attempt at polypectomy with healing and scarring of the two layers, mucosa and submucosa, preventing their separation by fluid injection. There is a theoretic possibility that injection through a malignant tumor may cause tracking of cancer cells into and even through the bowel wall. The risk of this happening is minimal, with experience gained from direct percutaneous needle aspiration of malignant tumors in other sites

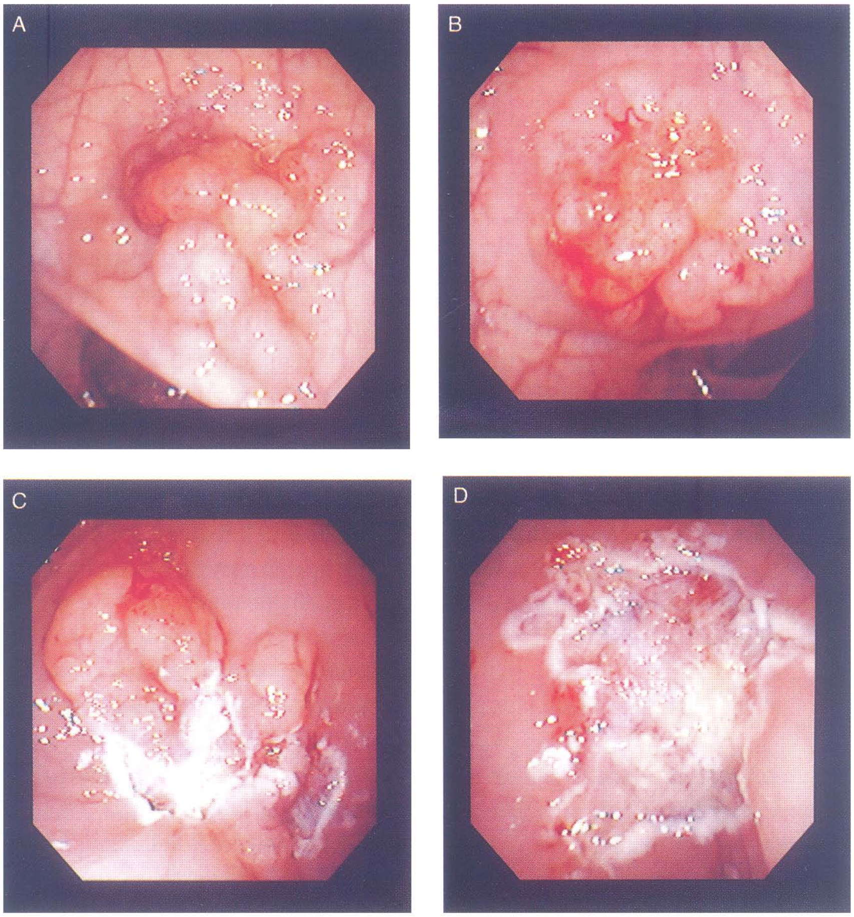

FIGURE 2 A. Flat polyp in right colon. B. Elevation following SIP technique with saline. C. Partial polypectomy. D. Total polypectomy completed in 11 min.

throughout the body. In the latter instances, the risk of tumor tracking is 1 in 10,000 to 1 in 20,000 cases [37].

Parenthetically, it seems that any tumor which can be elevated with submucosal injection of fluid may be totally removed by endoscopic resection, even if invasive cancer is found on tissue examination. The ability to elevate a tumor indicates that there is only a limited degree of fixation to the submucosal layer, with the possibility of complete removal.

It is permissible to remove a much larger piece with this technique than one would ordinarily resect when in the right colon without a "cushion" of fluid. The pieces should probably not be larger than 2 cm in diameter [34]. With the fluid as protection against deep thermal tissue injury, it is even possible to fulgurate the base of the polyp resection site. The devices used for fulguration include a hot biopsy forceps, the tip of the snare, the argon plasma coagulator, or any other thermal device which delivers heat to the residual polyp site.

Aspiration of air during attempted snare capture of the elevated polyp will result in an easier encirclement.

### Tenting the Polyp

After the wire is seated securely around the polyp, the sheath should be lifted slightly away from the wall, tenting it toward the lumen to elevate the polyp from the submucosa [38]. This will further limit the depth of injury when current is applied because the local zone of heating has a lessened chance of damaging the muscularis propria and serosa because the layers are pulled away from each other.

### Treating the Base After Polypectomy

If a sessile polyp is removed in piecemeal fashion, the base may be somewhat irregular. If tiny fragments of tissue are seen at the base, a repeat examination after four to twelve weeks may reveal that the polyp has completely disappeared, since thermal energy delivered during polypectomy may slough the remnants. If visible adenoma remains at the time of initial polypectomy, fulguration may be accomplished with a variety of thermal devices, including the argon plasma coagulator, a Bicap, heater probe, current applied to the barely extended snare wire, or a hot biopsy forceps [39]. In spite of all attempts to totally remove large sessile polyps (over 3 cm in diameter) when the polyp appears to have been completely visually removed with the snare, there is a 50% probability that there will be residual or recurrent adenoma at the site of original resection on the follow-up examination. If there is visible residual adenoma left at the polypectomy site, there is no possibility of total involution and residual polyp will be present on the follow-up examination. However, if visible residual adenoma is immediately treated with the argon plasma coagulator, the risk of residual adenoma then falls to 50% [40] (Fig. 3). Most of these recurrences can be subsequently endoscopically resected at the follow-up examination.

### PROBLEMS DURING POLYPECTOMY

### Position of the Polyp

To capture a polyp, one of the most important factors is that it be in proper position relative to the tip of the colonoscope. An attempt should be made to bring all polyps into the five o'clock position to facilitate snare placement [41]. This can usually be accomplished by rotation of the scope to reposition the face of the scope in relation to the adenoma.

Advantageous positioning may be best accomplished when the colonoscope shaft is straight, because a straight instrument transmits torque to the tip, whereas a loop in the shaft tends to absorb rotational motions applied to the scope. With a loop in the scope, the dial controls may no longer work effectively to turn the instrument tip because the cables which transmit motion are maximally stretched by the loop. These two negative forces,

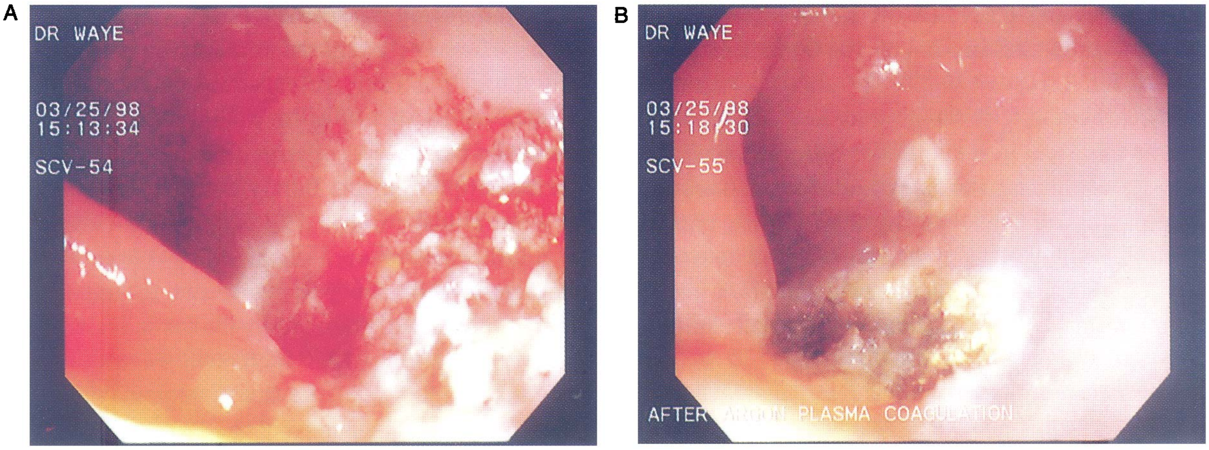

FIGURE 3 A. Base of polypectomy site with small fragment of residual adenoma, seen as multiple tiny elevations. B. Base following treatment with the argon plasma coagulator.

the inability to torque effectively and the loss of cable-controlled tip deflection, combine to create an extremely difficult situation when attempting to maneuver the snare into position around a polyp. Maneuvering can be made considerably easier by passing the scope far beyond the polyp, even to the cecum (and thus visualize the rest of the colon) and attempt capture during the withdrawal phase of the examination. As the scope is withdrawn, the loops are removed and the polyp which proved difficult to position during intubation may be quite easily ensnared because both torque and tip deflection are responsive when the shaft is straight.

An additional consideration to shift a polyp into a more favorable position is to change the patient's position or apply abdominal pressure. Polyps partially hidden behind folds may come more prominently into view as the patient's position is altered. Polyps submerged in a pool of fluid can be rotated into a drier field by turning the patient so that fluid flows away from the base.

### When to Remove the Polyp

Small polyps (less than 5 mm) in any position that are visualized during intubation should be removed at that time [41], since it may be difficult to find them again upon withdrawal of the instrument. If a medium-sized pedunculated polyp (up to 2 cm) is encountered during intubation, is in excellent position, and looks like it will be easy and straightforward to lasso, it is wise to perform polypectomy at that time since it may not be in such good position on withdrawal. There is no risk involved in passing the instrument beyond a fresh polypectomy site and performing total intubation of the colon.

### Rotatable and Mini Snares

Rotatable snares are considered unnecessary by most endoscopists. With the wire loop extended, the combination of torque on the shaft of the colonoscope and rotation of the dial controls affords much the same effect as snare wire rotation.

The standard regular-sized polypectomy snare may not be able to capture a small polyp in a difficult and "tight" location where there is not a sufficient distance for the wire loop to open sufficiently wide to be placed over a polyp. A problem with the standard snare is that it must be completely extended to its full length of 6 cm in order for the loop to completely expand. During colonoscopy, it often occurs that the wire loop can only be extended a few centimeters beyond the scope because of a tight bend or because the tip of the loop impacts on an adjacent wall of the colon. When the snare loop cannot be fully extended, the two partially open parallel wires may not sufficiently spread apart to enable polyp capture. In this circumstance, a "mini" snare 3 cm in length and 1 cm in width is extremely valuable [41,42]. This snare will open fully when extended only 3 cm beyond the sheath making it useful in areas where multiple bends are present (such as in the sigmoid narrowed with diverticulosis), or when polyps are located in the trough between intrahaustral folds. Since the vast majority of colon polyps are less than 1 cm in diameter, they are within the capability of this mini-snare.

### The Difficult Sigmoid

Even after total colonoscopy has been performed and the colonoscope has been straightened, there may still be difficulty in the sigmoid colon when attempting to capture a polyp because of narrowing by diverticula and thickened hypertrophic folds. A standard upper intestinal gastroscope has been demonstrated to be of benefit [43–45]. The major attributes of the gastroscope are that it has a tighter bending radius of the tip than does a colonoscope and the tip beyond the bending portion is shorter in length. This will frequently allow easy snare positioning in the same location where the colonoscope was both cumbersome and difficult. There is a growing awareness among endoscopists that gastroscopes can easily and readily be used in the colon to intubate difficult and narrowed segments, to be passed through strictures, and to render a previously inaccessible polyp more readily manageable.

### Clamshell Polyps

Large sessile polyps wrapped around a fold in a "clamshell" fashion usually permit the distal portion to be readily removed, but resection of the portion on the far side of the fold (proximal) may be considerably more difficult. This type of polyp is often located in the right colon and should be removed in piecemeal fashion. Although it would be ideal to resect the total polyp at one session, it may only be possible to remove the portion nearest to the scope, leaving some of the polyp on the far side of the fold for an interval resection. Subsequent scarring may flatten the polypectomy site, bringing the residual polyp into a favorable location for subsequent polypectomy. If it is elected to attempt total polypectomy at the first session, the stiffness of the plastic snare catheter can be used as a probe. After endoscopic transection of the portion closest to the scope, and with the loop extended, the tip of the catheter can be positioned on the ridge of the fold in the polypectomy site where a portion of the polyp has just been removed. By a combination of torque and rotation of the large control knob, downward pressure on the ridge at the site of the polypectomy divot will often depress it sufficiently so that a portion of the residual adenoma will extend into the loop permitting capture under direct vision. Several such attempts may be employed during piecemeal polypectomy. Pushing on a fresh polypectomy site in this manner is not associated with any adverse results.

An alternative technique for removal of a polyp located on the far side of a fold is to perform a U-turn maneuver. With standard instruments, this can only be accomplished in the cecum, ascending colon and transverse colon. It is difficult but not impossible to resect a polyp in a U-turn mode because the tip deflection responses are opposite to those usually expected.

### Polyp Size

The size of the polyp is an obvious reason for difficulty with endoscopic resection. Most polyps are less than 1 cm in diameter, a size that should be well within the resection capability of any trained colonoscopist. Twenty percent of polyps are larger than 10 mm in diameter and only 1% greater than 35 mm in size. When polyps in the left colon grow to become larger than 1 cm in diameter, it is common for peristalsis to pull on the lesion, forming a tubular pedicle from the surrounding mucosa. A polyp of the same size in the right colon has a tendency to remain sessile, with a broad-based attachment.

### Carpet Polyps

In spite of the knowledge and skill of modern endoscopists, not all colon polyps can be successfully removed with a colonoscope. Among these are carpet-like polyps which extend over several centimeters. An attempt can be made to fulgurate the surface of such polyps with the shank of the monopolar biopsy forceps, a Bicap probe, a laser, or the argon plasma coagulator. A helpful maneuver to be considered when the lesion appears too flat to capture with the snare loop is to aspirate air from the colon with the snare device in place. This will collapse the distended colon, causing the colon wall and the polyp to fold up, rendering capture relatively easy so that piecemeal type resection is possible. Alternate possibilities include submucosal injection of fluid to elevate the polyp for safer transection and use of a two-channel colonoscope where a forceps can be passed through one channel to grasp the polyp over which the opened snare has been positioned. Once the forceps lifts up the polyp, the snare is tightened to capture the polyp.

### Retrieval of Multiple Polyps or Fragments

Single polyps or a single fragment may be retrieved by suction into the tip of the colonoscope or captured with a snare and removed with the scope. When several small pieces remain in the colon, they may be suctioned into a collection device (trap). Two larger pieces may be captured in a small loop, but this maneuver requires considerable skill and patience. Several polyps or many fragments removed from the left colon or rectum may require several passes of the colonoscope with retrieval by suction or a snare. This is not feasible when several pieces are in the right colon, but two devices are useful in this situation. Dormia baskets with three or more wire arms are available, or a Roth basket (US Endoscopy Co., Mentor, Ohio, USA) may be employed. This retrieval basket is a special net fastened to a non-conductive snare loop. It is capable of repeated opening and capture of several fragments, following which the colonoscope and basket are removed.

### SITE LOCATION

In the era when laparoscopic-assisted surgical colonic resection is becoming as well accepted as primary colonoscopy, there is even greater urgency to have precise lesion location, since the laparoscopist does not have the capability of palpating the colon between the fingers at exploratory laparotomy [46]. For the laparoscopist, it is of great importance to have an easily visible marker which can be seen through the telescopic lens of the laparoscope. It is not acceptable for the endoscopist to state that "a lesion is in the transverse colon," since a more specific localization is needed to avoid a subsequent open surgery to find the lesion.

Precise location of the tip position during colonoscopy is equally important when there is a need to relocate a lesion or an area of the colon at a later point in time. It may be desirable to know the precise site at which a polyp was removed in piecemeal fashion so that the area can be readily identified at the next follow-up colonoscopy, especially if the polyp were on the back-side of a fold or just around a difficult bend of the colon. Even under circumstances when open laparotomy is to be performed, site identification becomes necessary when a specific portion of the large bowel requires resection and the lesion may not be readily apparent by visual or palpatory exploration. Following endoscopic removal of a malignant adenoma, the site may heal completely in eight weeks, and a locator mark may assist both the surgeon and the pathologist in identifying the place where the lesion had been.

### Magnetic Imaging

New methods of inductive sensing with a low-intensity magnetic field may aid in the moment-to-moment localization of the tip of the fiberoptic colonoscope as it progresses through the colon. The magnetic forces are attracted to sensors within the sheath of the colonoscope (or to sensors on a wand-like device inserted into the biopsy channel). Two different techniques have been developed in England, and will soon be ready for clinical trials

[47,48]. These methods have replaced such devices as metal detectors for localization of the instrument tip [49]. Unlike a fluoroscopic image which demonstrates both the scope and air in the colon as a contrast media, the electro-magnetic field method only shows the colonoscope itself, but is capable of a three-dimensional format. This technique may be of benefit in localizing the site of a colonic tumor or polyp, and is undergoing clinical trials for this purpose.

### Intraoperative Endoscopy

It is possible to localize the site of a tumor, or a resected polypectomy site, by performing intraoperative colonoscopy [50–52]. This technique has been avoided by most endoscopists because of the need to perform an endoscopic examination in the operating room with all the constraints of positioning the patient, handling the scope, and trying to use maneuvers such as torque and straightening techniques with the abdomen open. The amount of air insufflated for colonoscopy can create problems with surgical techniques once the endoscopist has completed the necessary localization. Because the site of a polypectomy may heal within a few weeks, there is a possibility that a polypectomy site may not be seen during an intraoperative endoscopy.

### Injectable Marker

The ideal method for lesion localization is to have an easily identifiable marker which will immediately draw the attention of the surgeon or endoscopist [53]. This can be achieved with injection of dye solutions. An experimental study demonstrated that, of eight different dyes injected into the colon wall in experimental animals, only two persisted for more than 24 h [54]. These were indocyanine green and India ink. The indocyanine green was visible up to seven days after injection, and it is known that India ink is a permanent marker which lasts for the life of the patient by virtue of submucosal injection of carbon particles. Other dyes, such as methylene blue, indigo carmine, toluidine blue, lymphazurine, hematoxylin, and eosin, all were absorbed within 24 h leaving no residual stain at the injection site. Indocyanine green is approved by the FDA for human use, but India ink has not been so approved. Indocyanine green is not associated with any significant tissue reaction, and is relatively non-toxic [54]. It provides excellent staining of the serosal surface and draining lymphatics for up to seven days following its injection.

Clinical experience with indocyanine green tattoo in twelve patients demonstrated that the dye was easily visualized on the serosal surface of the colon at surgery within 36 h following injection [55]. The problem with a marker having such a relatively short time span is that the decision to operate after removal of a malignant polyp may require a few weeks, with slide reviews and multiple consultations. An injection at the time of polypectomy will have disappeared whereas the site will become more difficult to localize with the passage of time.

### Carbon Particle Injection

Most experience with dye injection technique has been accumulated with India ink as a permanent marker [56,57]. The stain lasts for at least ten years with no diminution in intensity at that duration. A permanent marker may be worthwhile for several reasons. A lesion requiring surgery may be injected and, for clinical reasons, surgery may be postponed for several weeks at which time a vital dye such as indocyanine green will have been absorbed, leaving the operating surgeon with no visible evidence of its having been injected. Sometimes it is desirable to mark the site of a resected polyp for subsequent endoscopic localization when it is anticipated that the area will be difficult to find on a follow-up examination, especially when the lesion is located around a fold or behind a haustral septum. A stain with a permanent marker such as India ink will draw immediate attention to the site, enabling a more accurate and complete assessment. For the surgeon, a locator stain will aid immeasurably the efforts to seek and resect an area of the bowel containing the site of the lesion. When the lesion is relatively small, such as a flat cancer or a previously endoscopically resected malignant polyp which requires surgical resection, the site may not be evident from the serosal surface and may not even be palpable. If the area to be resected is in a redundant sigmoid colon or near the splenic flexure, it may be impossible to locate by either visual means or by palpation. Occasionally, even large lesions may not be palpable by the surgeon if they are soft and compressible [51]. As previously mentioned, visible marking can assist in precise surgical intervention for laparoscopic-assisted colon resections, or clips may be detected by an ultrasound probe.

There have been reported complications with India ink injection, but these are relatively rare [58,59]. The complications may in part be related to the wide variety of organic and inorganic compounds contained in the ink solution, such as carriers, stabilizers, binders, and fungicides [60]. It is possible that the toxic properties of India ink may be partially ameliorated by marked dilution of the ink. Ink diluted to 1 : 100 with saline produces as dark a spectrophotometric pattern as undiluted India ink, and in clinical tests, the tattoo made by 1 : 100 diluted India ink is readily visible by the endoscopist and by the operating surgeon. A small volume injection may increase the safety of the procedure [61,62].

India ink is black drawing ink made with carbon particles. An intracavity injection is not a clinical problem [55,63]. but can scatter black carbon particles around the abdominal cavity, which may be somewhat disconcerting for the surgeon.

### Circumferential Injections

Since the colonoscopist cannot know which portion of the bowel is the superior aspect, multiple injections should be made circumferentially in the wall around a lesion to prevent a single injection site from being located in a "sanctuary" site, hidden from the eyes of the surgeon as the abdomen is opened with the patient lying supine [64]. Each injection should be of sufficient volume to raise a bluish bleb within the mucosa at the injection site

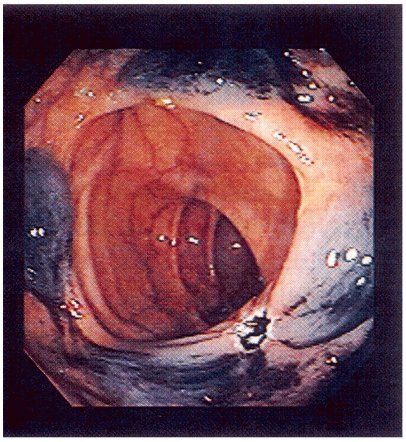

FIGURE 4 Circumferential marking of polypectomy site with carbon particle injection.

(Fig. 4). The injection volume may vary from 0.1 to 0.5 ml. If injections are made a few centimeters from the lesion, the surgeon should be informed whether the injections are proximal or distal to the site. With the 1 : 100 dilution of India ink, endoscopic visualization is still possible should some of the ink spill into the lumen, whereas, with the more concentrated solutions, the endoscopic picture becomes totally black when ink covers the bowel walls [63]. Several reports have attested as to its safety as well as its efficacy [58,65,66].

### *References*

[1] Valentine, J.F. Double-channel endoscopic polypectomy technique for the removal of large pedunculated polyps. *Gastrointest. Endosc.* 1998; **48**: 314–316.

[2] Akahoshi, K., Kojima, H., Fujimaru, T. *et al.* Grasping forceps assisted endoscopic resection of large pedunculated GI polypoid lesions. *Gastrointest. Endosc.* 1999; **50**: 95–98.

[3] Kawamoto, K., Yamada, Y., Furukawa, N. *et al.* Endoscopic submucosal tumorectomy for gastrointestinal submucosal tumors restricted to the submucosa: a new form of endoscopic minimal surgery. *Gastrointest. Endosc.* 1997; **46**: 311–317.

[4] Waye, J.D. New methods of polypectomy. *Gastrointest. Endosc. Clin. N. Am.* 1997; **7**: 413–422.

[5] Johanns, W., Luis, W., Janssen, J. *et al.* Argon plasma coagulation (APC) in gastroenterology: experimental and clinical experiences. *Eur. J. Gastroenterol. Hepatol.* 1997; **9**: 581–587.

[6] Farin, G. and Grund, K.E. Technology of argon plasma coagulation with particular regard to endoscopic applications. *Endosc. Surg. Allied Technol.* 1994; **2**: 71–77.

[7] Grund, K.E., Storek, D. and Farin, G. Endoscopic argon plasma coagulation (APC) first clinical experiences in flexible endoscopy. *Endosc. Surg. Allied Technol.* 1994; **2**: 42–46.

[8] Storek, D., Grund, K.E., Gronbach, G. *et al.* Endoscopic argon gas coagulation – initial clinical experiences. *Z. Gastroenterol.* 1993; **31**: 675–679.

[9] Rey, J.F. and Marek, T.A. Endo-loop in the prevention of the post-polypectomy bleeding: preliminary results. *Gastrointest. Endosc.* 1997; **46**: 387–389.

[10] Waye, J.D., Lewis, B.S. and Yessayan, S. Colonoscopy: a prospective report of complications. *J. Clin. Gastroenterol.* 1992; **15**: 347–351.

[11] Matsushita, M., Hajiro, K., Takakuwa, H. *et al.* Ineffective use of a detachable snare for colonoscopic polypectomy of large polyps. *Gastrointest. Endosc.* 1998; **47**: 496–499.

[12] Ellis, K.K. and Fennerty, M.B. Marking and identifying colon lesions. Tattoos, clips, and radiology in imaging the colon. *Gastrointest. Endosc. Clin. N. Am.* 1997; **7**: 401–411.

[13] Nagasu, N. and DiPalma, J.A. Bleeding ulcer: inject or clip? *Am. J. Gastroenterol.* 1998; **93**: 1998.

[14] Hachisu, T., Yamada, H., Satoh, S. *et al.* Endoscopic clipping with a new rotatable clip-device and a long clip. *Dig. Endosc.* 1996; **8**: 127–133.

[15] Uno, Y., Satoh, K., Tuji, K. *et al.* Endoscopic ligation by means of clip and detachable snare for management of colonoscopic postpolypectomy hemorrhage. *Gastrointest. Endosc.* 1999; **49**: 113–115.

[16] Iida, Y., Miura, S., Munemoto, Y. *et al.* Endoscopic resection of large colorectal polyps using a clipping method. *Dis. Colon Rectum* 1994; **37**: 179–180.

[17] Cohen, L.B. and Waye, J.D. Treatment of colonic polyps – practical considerations. *Clin. Gastroenterol.* 1986; **15**: 359.

[18] McNally, D.O., DeAngelis, S.A., Rison, D.R. *et al.* Bipolar polypectomy device for removal of colon polyps. *Gastrointest. Endosc.* 1994; **40**: 489–491.

[19] Waye, J.D., Lewis, B.S., Frankel, A. *et al.* Small colon polyps. *Am. J. Gastroenterol.* 1988; **83**: 120–122.

[20] Peluso, F. and Goldner, F. Follow-up of hot biopsy forceps treatment of diminutive colonic polyps. *Gastrointest. Endosc.* 1991; **37**: 604–606.

[21] Woods, A., Sanowski, R.A., Wadas, D.D. *et al.* Eradication of diminutive polyps: a prospective evaluation of bipolar coagulation versus conventional biopsy removal. *Gastrointest. Endosc.* 1989; **35**: 536.

[22] Vanagunas, A., Jacob, P. and Vakil, N. Adequacy of "hot biopsy" for the treatment of diminutive polyps: a prospective randomized trial. *Am. J. Gastroenterol.* 1989; **84**: 383.

[23] Wadas, D.D. and Sanowski, R.A. Complications of the hot biopsy forceps technique. *Gastrointest. Endosc.* 1988; **34**: 32–37.

[24] Tappero, G., Gaia, E., DeFiuli, P. *et al.* Cold snare excision of small colorectal polyps. *Gastrointest. Endosc.* 1992; **38**: 310–313.

[25] Uno, Y., Obara, K., Zheng, P. *et al.* Cold snare excision is a safe method for diminutive colorectal polyps. *Tohoku J. Exp. Med.* 1997; **183**: 243–249.

[26] Geenen, J.E., Fleischer, D. and Waye, J.D. *Techniques in Therapeutic Endoscopy* (2nd edn.). New York: W.B. Saunders and Gower Medical Publishing, Inc., 1992.

[27] Waye, J.D. Techniques of polypectomy: hot biopsy forceps and snare polypectomy. *Am. J. Gastroenterol.* 1987; **82**: 615–618.

[28] Tsuga, K., Haruma, K., Fujimura, J. *et al.* Evaluation of the colorectal wall in normal subjects and patients with ulcerative colitis using an ultrasonic catheter probe. *Gastrointest. Endosc.* 1998; **48**: 477–484.

[29] Karita, M., Tada, M., Okita, K. *et al.* Endoscopic therapy for early colon cancer: the strip biopsy resection technique. *Gastrointest. Endosc.* 1991; **37**: 128–132.

[30] Karita, M., Tada, M. and Okita, K. The successive strip biopsy partial resection technique for large early gastric and colon cancers. *Gastrointest. Endosc.* 1992; **38**: 174–178.

[31] Karita, M., Cantero, D. and Okita, K. Endoscopic diagnosis and resection treatment for flat adenoma with severe dysplasia. *Am. J. Gastroenterol.* 1993; **88**: 1421–1423.

[32] Uchikawa, H., Hirao, M., Yamaguti, O. *et al.* Endoscopic mucosal resection combined with local injection of hypertonic saline epinephrine solution for early colorectal cancers and other tumors. *Gastrointest. Endosc.* 1992; **34**: 1871–1878.

[33] Shirai, M., Nakamura, T., Matsuura, A. *et al.* Safer colonoscopic polypectomy with local submucosal injection of hypertonic saline-epinephrine solution. *Am. J. Gastroenterol.* 1994; **89**: 334–338.

[34] Kanamori, T., Itoh, M., Yokoyama, Y. *et al.* Injection-incision-assisted snare resection of large sessile colorectal polyps. *Gastrointest. Endosc.* 1996; **43**: 189–193.

[35] Waye, J.D. Saline injection colonoscopic polypectomy: editorial. *Am. J. Gastroenterol.* 1994; **89**: 305–306.

[36] Uno, Y. and Munakata, A. The non-lifting sign of invasive colon cancer. *Gastrointest. Endosc.* 1994; **40**: 485–489.

[37] Schiano, T.D., Pfister, D., Harrison, L. *et al.* Neoplastic seeding as a complication of percutaneous endoscopic gastrostomy. *Am. J. Gastroenterol.* 1994; **89**: 131–133.

[38] Waye, J.D. Polyps large and small. Editorial. *Gastrointest. Endosc.* 1992; **38**: 391–392.

[39] Walsh, R.M., Ackroyd, F.W. and Shelito, P.C. Endoscopic resection of large sessile colorectal polyps. *Gastrointest. Endosc.* 1992; **38**: 303–309.

[40] Zlatanic, J., Waye, J.D., Kim, P.S. *et al.* Large sessile colonic adenomas: use of argon plasma coagulator to supplement piecemeal snare polypectomy. *Gastrointest. Endosc.* 1999; **49**: 731–735.

[41] Waye, J.D. Endoscopic treatment of adenomas. *World J. Surg.* 1991; **15**: 14–19.

[42] McAfee, J.H. and Katon, R.M. Tiny snares prove safe and effective for removal of diminutive colorectal polyps. *Gastrointest. Endosc.* 1994; **40**: 301–303.

[43] Bat, L. and Williams, C.B. Usefulness of pediatric colonoscopes in adult colonoscopy. *Gastrointest. Endosc.* 1989; **35**: 329–332.

[44] Rogers, B.H.G. The use of small caliber endoscopes in selected cases increases the success rate of colonoscopy. *Gastrointest. Endosc.* 1989; **35**: 352.

[45] Kozarek, R.A., Botoman, V.A. and Patterson, D.J. Prospective evaluation of a small caliber upper endoscope for colonoscopy after unsuccessful standard examination. *Gastrointest. Endosc.* 1989; **35**: 333–335.

[46] Hancock, J.H. and Talbot, R.W. Accuracy of colonoscopy in localization of colorectal cancer. *Int. J. Colorectal Dis.* 1995; **10**: 140–141.

[47] Bladen, J.S., Anderson, A.P., Bell, G.D. *et al.* Non-radiological technique for three-dimensional imaging of endoscopes. *Lancet* 1993; **341**: 719–722.

[48] Williams, C., Guy, C., Gilles, D. *et al.* Electronic three-dimensional imaging of intestinal endoscopy. *Lancet* 1993; **341**: 724–725.

[49] Leicester, R.J. and Williams, C.B. Use of metal detector for localisation during fibresigmoidoscopy or limited colonoscopy. *Lancet* 1981; **2**: 232–233.

[50] Forde, K.A. and Cohen, J.L. Intraoperative colonoscopy. *Ann. Surg.* 1988; **207**: 231–233.

[51] Richter, R.M., Littman, L. and Levowitz, B.S. Intraoperative fiberoptic colonoscopy. Localization of nonpalpable colonic lesions. *Arch. Surg.* 1973; **106**: 228.

[52] Sakanoue, Y., Nakao, K., Shoji, Y. *et al.* Intraoperative colonoscopy. *Surg. Endosc.* 1993; **7**: 84–87.

[53] Hilliard, G., Ramming, K., Thompson Jr., J. *et al.* The elusive colonic malignancy. A need for definitive preoperative localization. *Am. Surg.* 1990; **56**: 742–744.

[54] Hammond, D.C., Lane, F.R., Welk, R.A. *et al.* Endoscopic tattooing of the colon: an experimental study. *Am. Surg.* 1989; **55**: 457–461.

[55] Hammond, D.C., Lane, F.R., Mackeigan, J.M. *et al.* Endoscopic tattooing of the colon: clinical experience. *Am. Surg.* 1993; **59**: 205–210.

[56] Ponsky, J.L. and King, J.F. Endoscopic marking of colon lesions. *Gastrointest. Endosc.* 1975; **22**: 42–43.

[57] Cohen, L.B. and Waye, J.D. Colonoscopic polypectomy of polyps with adenocarcinoma: when is it curative? In: Barkin, J.S. (Ed.) *Difficult Decisions in Digestive Diseases*. Chicago: Year Book Medical Publishers, 1989: pp. 528–535.

[58] Nizam, R., Siddiqi, N., Landas, S.K. *et al.* Colonic tattooing with India ink: benefits, risks, and alternatives. *Am. J. Gastroenterol.* 1996; **91**: 1804–1808.

[59] Gopal, D.V., Morava-Protzner, I., Miller, H.A.B. *et al.* Idiopathic inflammatory bowel disease associated with colonic tattooing with India ink preparation. *Gastrointest. Endosc.* 1999; **49**: 636–639.

[60] Lightdale, C.J. India ink colonic tattoo – blots on the record (edn.). *Gastrointest. Endosc.* 1991; **37**: 68–71.

[61] Poulard, J.B., Shatz, B. and Kodner, I. Preoperative tattooing of polypectomy site. *Endoscopy* 1985; **17**: 84–85.

[62] Shatz, B.A. Small volume India ink injections (letter to editor). *Gastrointest. Endosc.* 1991; 37: 649–650.

[63] Shatz, B.A. and Thavorides, V. Colonic tattoo for follow-up of endoscopic sessile polypectomy. *Gastrointest. Endosc.* 1991; **37**: 59–60.

[64] Hyman, N. and Waye, J.D. Endoscopic four quadrant tattoo for the identification of colonic lesions at surgery. *Gastrointest. Endosc.* 1991; **37**: 56–58.

[65] Shatz, B.A., Weinstock, L.B., Swanson, P.E. *et al.* Long-term safety of India ink tattoos in the colon. *Gastrointest. Endosc.* 1997; **45**: 153–156.

[66] McArthur, C.S., Roayaie, S. and Waye, J.D. Safety of preoperation endoscopic tattoo with India ink for identification of colonic lesions. *Surg. Endosc.* 1999; **13**: 397–400.

